# Supplementary material for: Mutational Asymmetries in the SARS-CoV-2 Genome May Lead to Increased Hydrophobicity of Virus Proteins
Source: Genes (Basel). 2021 May 27;12(6):826. doi: 10.3390/genes12060826 (PMC8227412; doi:10.3390/genes12060826)
Supplement: Supplementary file 1 [file genes-12-00826-s001.zip › Figure S1.pdf]

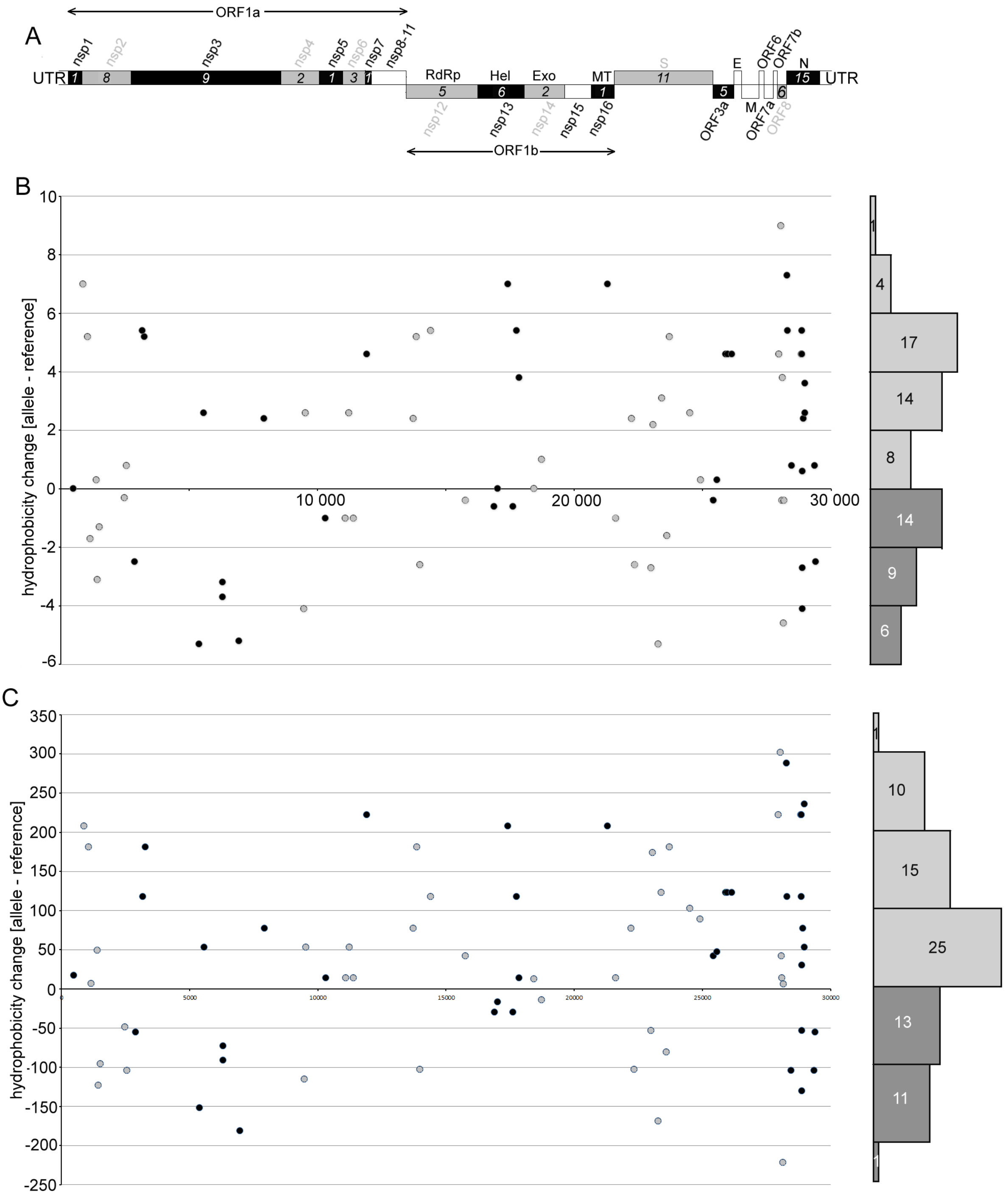

Figure S1. Character of amino acid hydrophobicity changes induced by nucleotide substitutions along the SARS-CoV-2 genome – comparison of two hydrophobicity scales. (A) A scheme of the SARS-CoV-2 genome organization. Numerals in italics indicate number of substitutions within each gene body. Genes with no detected substitution are in white. Nsp1-16 indicate genes for nonstructural proteins: RdRp = RNA-dependent RNA polymerase, Hel = helicase, Exo = 3'-to-5' exonuclease, Met = 2'-O-ribose methyltransferase. Structural proteins are represented by surphace glycoprotein (S), membrane glycoprotein (M) , nucleocapsid phospohoprotein (N) and envelope protein (E). (B, C) Characters of amino acid hydrophobicity changes computed using a hydrophobicity scales of Kyte and Doolittle [33] (B) and Palecz [34] (C). For better orientation, the values (circles) are shown in black or gray colors which assign them to the same colored (black and gray) alternating genes in panel A. Number of substitutions in individual hydrophobicity intervals is given on the right margin.
