# Supplementary material for: Mutational Asymmetries in the SARS-CoV-2 Genome May Lead to Increased Hydrophobicity of Virus Proteins
Source: Genes (Basel). 2021 May 27;12(6):826. doi: 10.3390/genes12060826 (PMC8227412; doi:10.3390/genes12060826)

Figure S2. Changes in amino acid hydrophobicity induced by different types of single nucleotide substitutions in different SARS-CoV-2 clades. Bar charts (A, C) and statistical evaluation (B, D) of shifts in cumulative hydrophobicity values. (A, B) – a hydrophobicity scale taken from [33], (C, D) – a hydrophobicity scale from [34]. Boxes – Q1 and Q3 quartiles. The vertical line inside the box marks the median. Note, extreme outliers corresponding to C>U, G>U and C>A substitutions. The outlying values are defined as follows: upper outlier = Q3 + 3*IQ, lower outlier = Q1 - 3*IQ, where IQ is the difference (interquartile) between the third (Q3) and first (Q1) quartile (Q3-Q1).

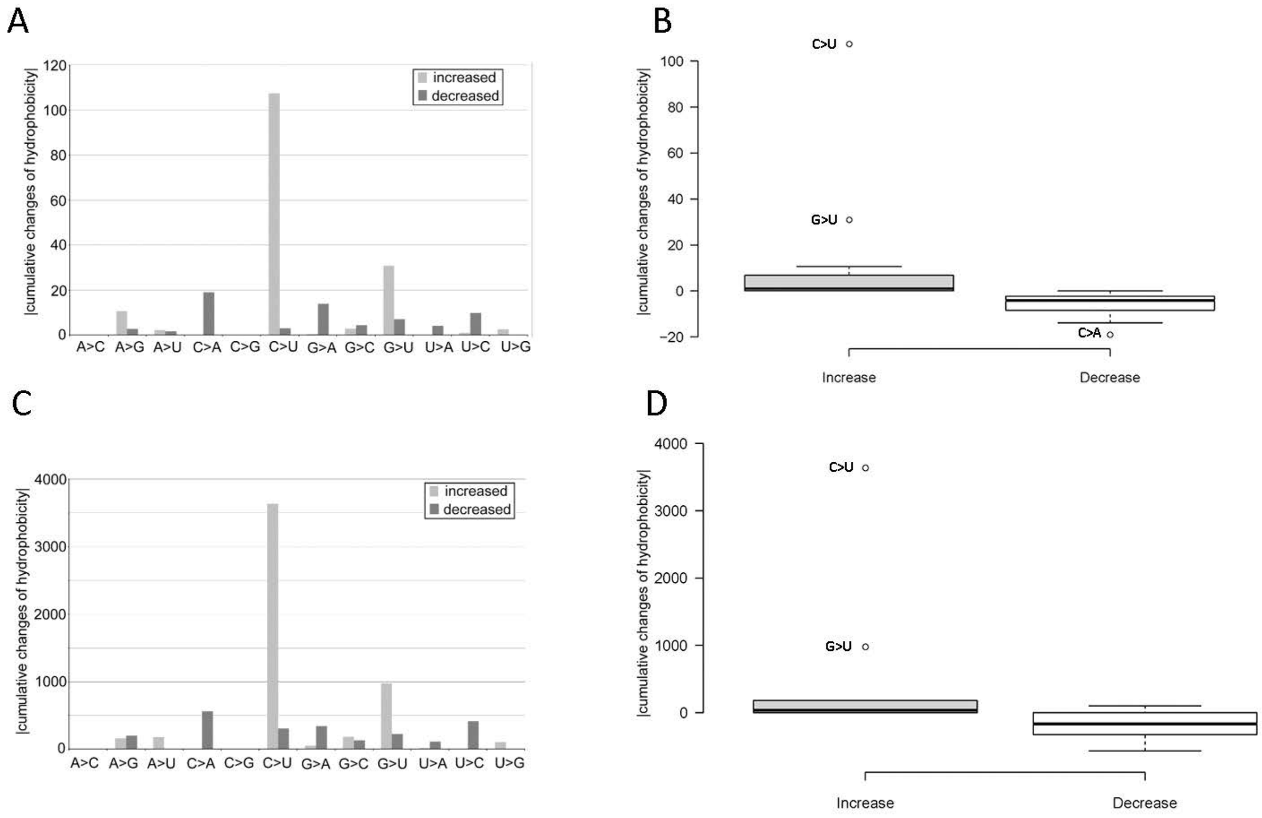

Supplement: Supplementary file 1 [file genes-12-00826-s001.zip › Figure S2.pdf]
