# Supplementary material for: Mutational Asymmetries in the SARS-CoV-2 Genome May Lead to Increased Hydrophobicity of Virus Proteins
Source: Genes (Basel). 2021 May 27;12(6):826. doi: 10.3390/genes12060826 (PMC8227412; doi:10.3390/genes12060826)

Figure S3. Relative frequency of synonymous/nonsynonymous SNVs in individual SARS-CoV-2 regions. Number above the columns indicate SNV counts.

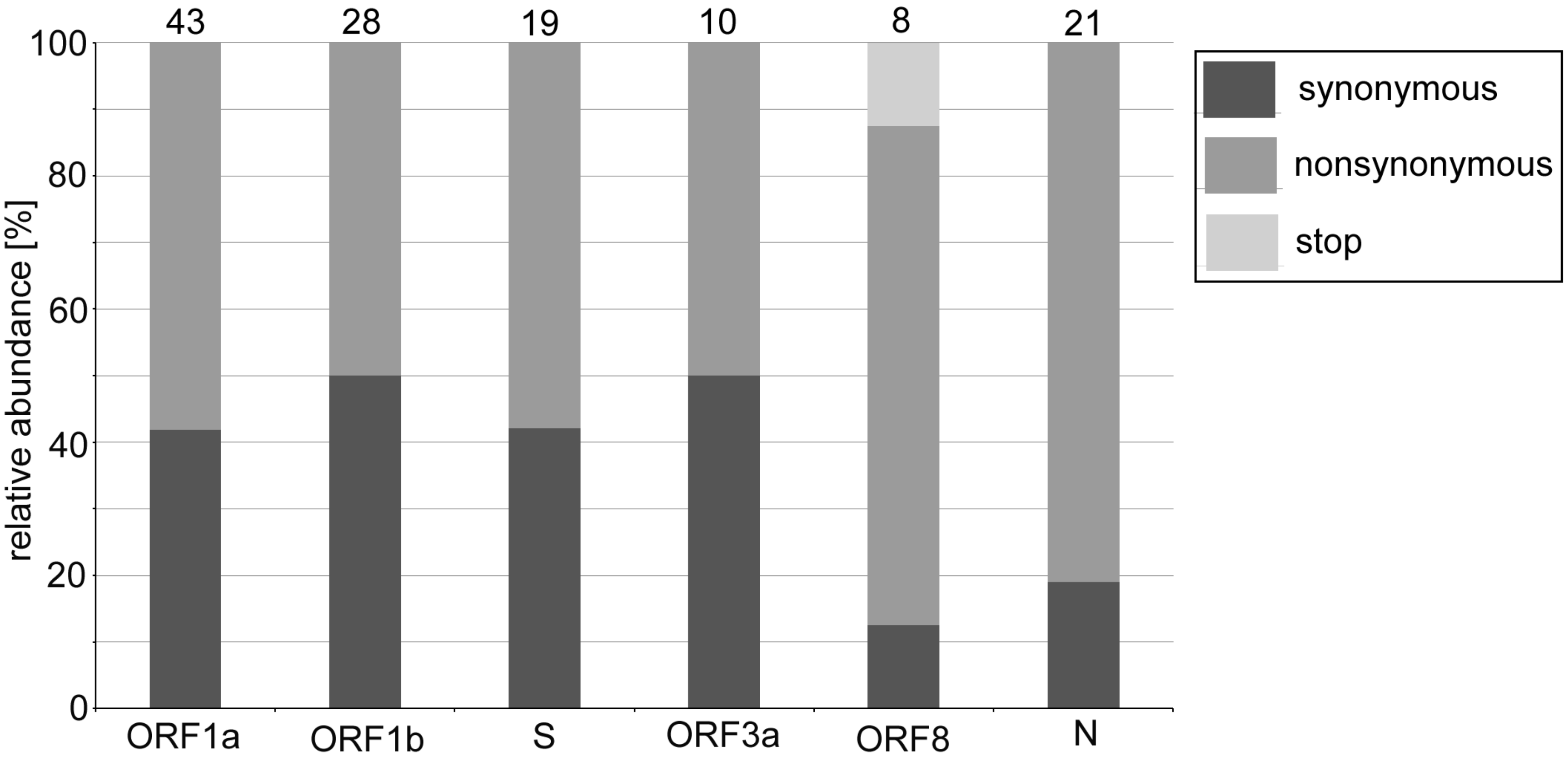

Supplement: Supplementary file 1 [file genes-12-00826-s001.zip › Figure S3.pdf]
