# Supplementary material for: Mutational Asymmetries in the SARS-CoV-2 Genome May Lead to Increased Hydrophobicity of Virus Proteins
Source: Genes (Basel). 2021 May 27;12(6):826. doi: 10.3390/genes12060826 (PMC8227412; doi:10.3390/genes12060826)

Figure S4. Computer prediction of antigenicity profiles of SARS-CoV-2 Spike proteins. (A) Profile of a protein derived from the reference genome. (B) Profile of the mutant protein containing frequently containing mutations in the GR lineage. Red and green arrows changes in antigenicity caused by Pro681His and Thr716Ile mutations. Both substitutions occur at comparable frequencies (20%) in the GR clade viruses.

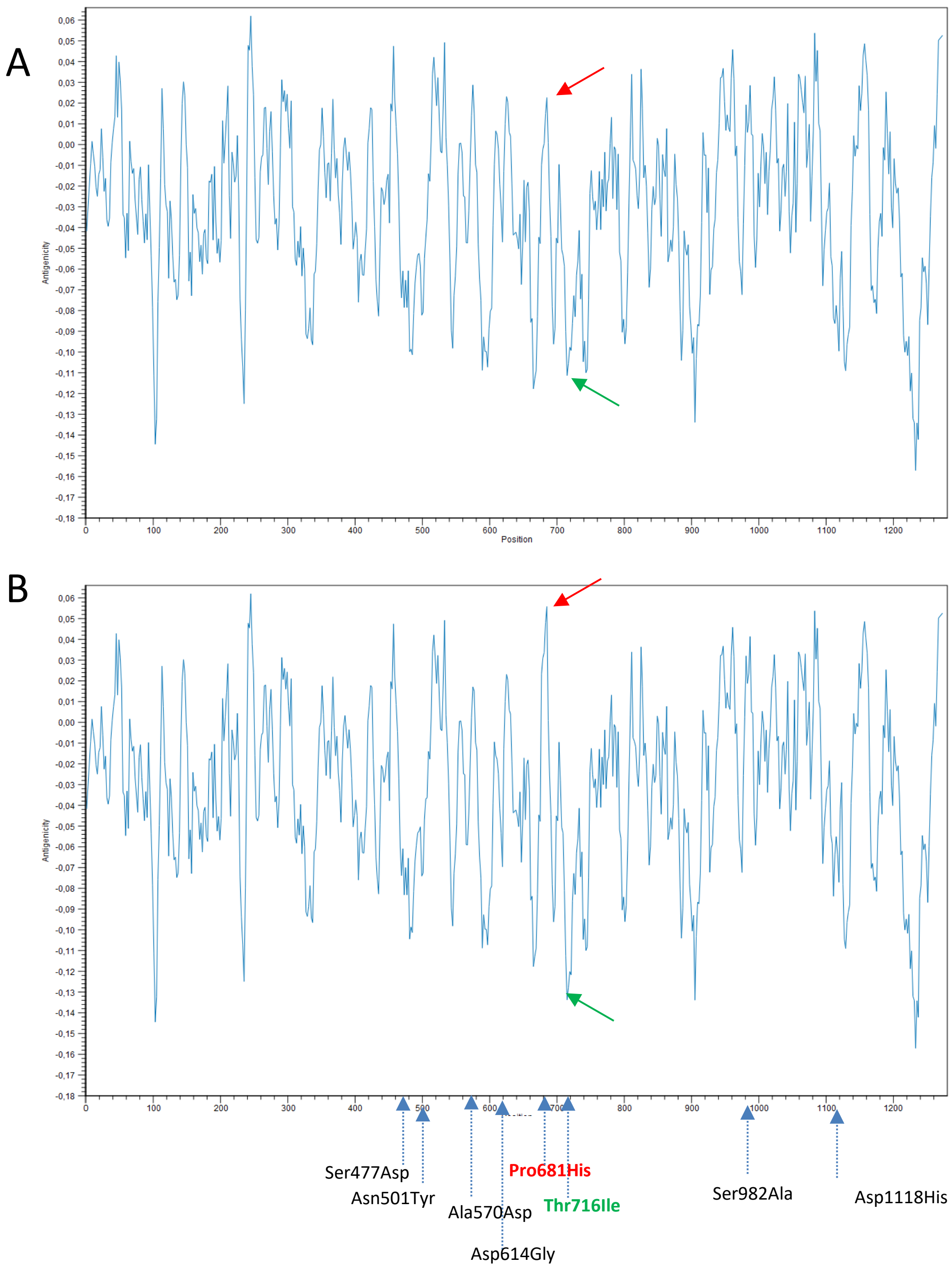

Supplement: Supplementary file 1 [file genes-12-00826-s001.zip › Figure S4.pdf]
