# Supplementary material for: Mutational Asymmetries in the SARS-CoV-2 Genome May Lead to Increased Hydrophobicity of Virus Proteins
Source: Genes (Basel). 2021 May 27;12(6):826. doi: 10.3390/genes12060826 (PMC8227412; doi:10.3390/genes12060826)

Figure S6. Alignment of selected genomes from the GR and G clades. The N-gene subregion (genomic position 28834-28888) encoding amino acids 186-209 is shown. The frequently occuring mutations are flagged. Dots – same nucleotides as in the reference. Note, multiple mutations within the same sequence are rare except of a conserved trinucleotide GGA>AAC substitution in the GR clade sequences.

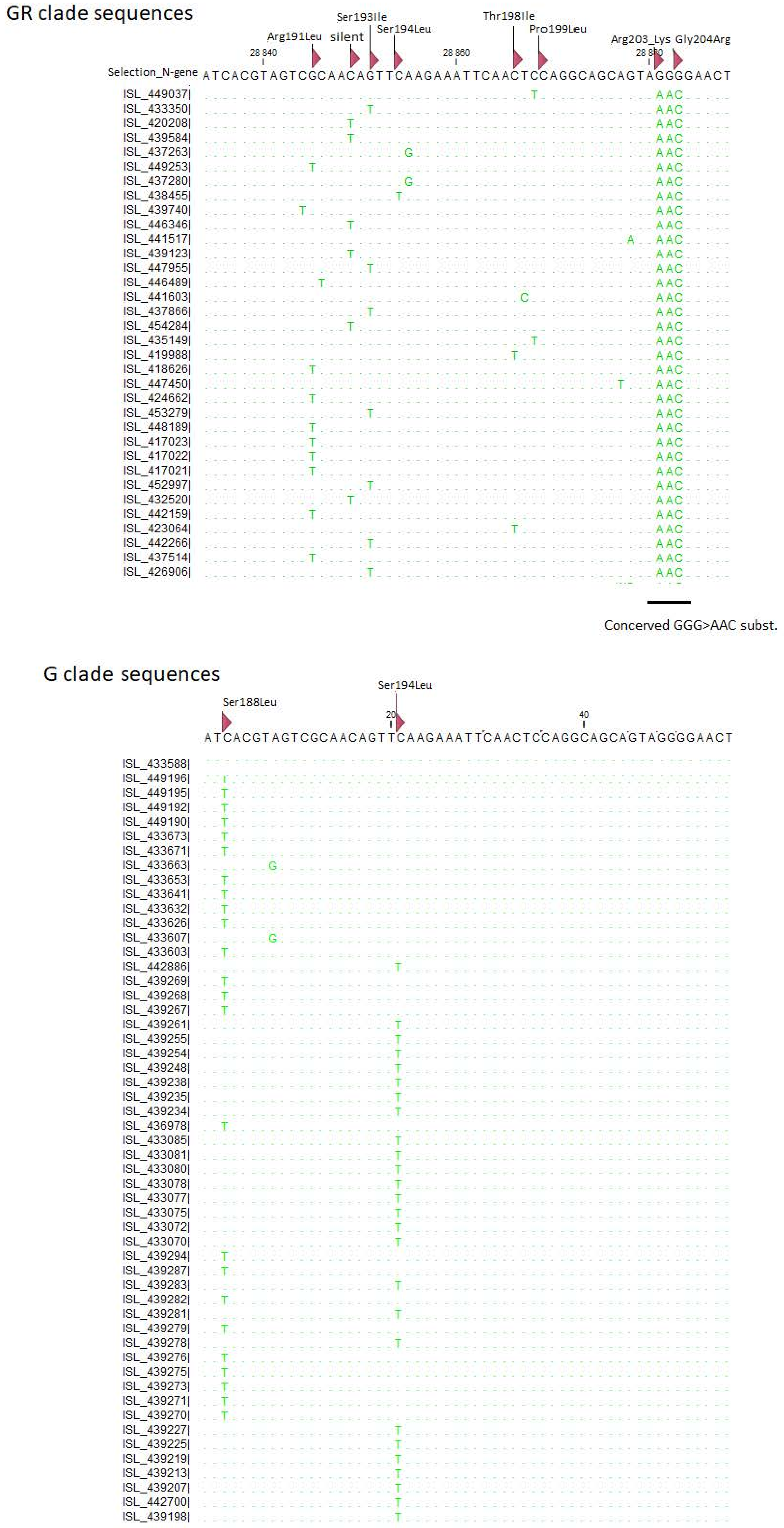

Supplement: Supplementary file 1 [file genes-12-00826-s001.zip › Figure S6.pdf]
