# Supplementary material for: Mutational Asymmetries in the SARS-CoV-2 Genome May Lead to Increased Hydrophobicity of Virus Proteins
Source: Genes (Basel). 2021 May 27;12(6):826. doi: 10.3390/genes12060826 (PMC8227412; doi:10.3390/genes12060826)
Supplement: Supplementary file 1 [file genes-12-00826-s001.zip › Table S1.pdf]

Table S1. Genome coordinates of SARS-CoV-2 proteins

ORF	Protein ID	start nt	end nt	protein name and description
ORF1a	YP_009725297.1	266	805	nsp1; leader peptide
	YP_009725298.1	806	2719	nsp2; replicase
	YP_009725299.1	2720	8554	nsp3; papain-like proteinase
	YP_009725300.1	8555	10054	nsp4; contains transmembrane domain TM2
	YP_009725301.1	10055	10972	nsp5; 3C-like proteinase
	YP_009725302.1	10973	11842	nsp6; putative transmembrane domain
	YP_009725303.1	11843	12091	nsp7; forms a complex with nsp12
	YP_009725304.1	12092	12685	nsp8; forms a complex with nsp12
	YP_009725305.1	12686	13024	nsp9; ssRNA-binding protein
	YP_009725306.1	13025	13441	nsp10; CysHis rich protein
	YP_009725312.1	13442	13480	nsp11; identical to the first segment of Nsp12
ORF1b	YP_009725307.1	13442	16236	nsp12; RdRp. Forms a complex with NSP7 and NSP8
	YP_009725308.1	16237	18039	nsp13; helicase
	YP_009725309.1	18040	19620	nsp14; 3'-to-5' exonuclease
	YP_009725310.1	19621	20658	nsp15; endoRNAse
	YP_009725311.1	20658	21552	nsp16; 2'-O-ribose methyltransferase
ORF_S	YP_009724390.1	21563	25384	sp; surphace glycoprotein S (spike)
ORF3a	YP_009724391.1	25393	26220	ORF3a protein
ORF4	YP_009724392.1	26245	26472	sp; envelope protein E
ORF5	YP_009724393.1	26523	27191	sp; membrane glycoprotein M
ORF6	YP_009724394.1	27202	27387	ORF6 protein
ORF7a	YP_009724395.1	27394	27759	ORF7a protein
ORF7b	YP_009725318.1	27756	27887	ORF7b protein
ORF8	YP_009724396.1	27894	28259	ORF8 protein
ORF9	YP_009724397.2	28274	29533	sp; nucleocapsid phospohoprotein N
ORF10	YP_009725255.1	29558	29674	ORF10 protein

nsp - non structural protein
sp - structural protein
RdRp - RNA-dependent RNA polymerase (frame shift protein)
