# Supplementary material for: Mutational Asymmetries in the SARS-CoV-2 Genome May Lead to Increased Hydrophobicity of Virus Proteins
Source: Genes (Basel). 2021 May 27;12(6):826. doi: 10.3390/genes12060826 (PMC8227412; doi:10.3390/genes12060826)
Supplement: Supplementary file 1 [file genes-12-00826-s001.zip › Table S10.pdf]

Table S10. Relationship between biochemical properties of amino acids and nucleotide compositions of corresponding codons

**Amino acid**			**Codons**	**[U] - uracil content** [%]			**[A] – adenine content** [%]		
**Character**	**aa**	**Hydr.**[1)]		pos. 1	pos. 2	pos. 3	pos. 1	pos. 2	pos. 3
strongly hydrophob.	Ile	4.50	AUU. AUC. AUA	0	100	33	100	0	33
	Leu	3.80	CUU. CUC. CUA. CUG. UUA. UUG	33	100	17	0	0	33
	Phe	2.80	UUU. UUC	100	100	50	0	0	0
	Val	4.20	GUU. GUC. GUA. GUG	0	100	25	0	0	25
	mean	3.83		33.3	100	31.3	25.0	0.0	22.8
strongly hydrophilic	Asn	-3.50	AAU. AAC	0	0	50	100	100	0
	Asp	-3.50	GAU. GAC	0	0	50	0	100	0
	Arg	-3.50	CGU. CGC. CGA. CGG. AGA. AGG	0	0	17	33	0	33
	Lys	-3.90	AAA. AAG	0	0	0	100	100	50
	Gln	-3.50	CAG. CAA	0	0	0	0	100	50
	Glu	-3.50	GAA. GAG	0	0	0	0	100	50
	mean	-3.57		0.0	0.0	19.5	38.8	83.3	30.5

[1] Hydrophobicity values are according to [33].
