# Supplementary material for: Mutational Asymmetries in the SARS-CoV-2 Genome May Lead to Increased Hydrophobicity of Virus Proteins
Source: Genes (Basel). 2021 May 27;12(6):826. doi: 10.3390/genes12060826 (PMC8227412; doi:10.3390/genes12060826)
Supplement: Supplementary file 1 [file genes-12-00826-s001.zip › Table S2.pdf]

Table S2. List of SARS-CoV-2 single nucleotide variants (SNV), their genomic position, character, occurrence in clades and corresponding amino acids changes

Region	Gene	Coordinate	Clade(s)	SNV	Context	S/N*	Amino acid change
5'UTR		204	GV	G>U	CGU		
		222	GV	C>U	UCA		
		241	O,G,GH,GR,GV	C>U	UCG		
ORF1a	nsp1	445	GV	U>C	UUU	S	Val60Val
		490	S	U>A	AUG	N	Asp75Glu
		514	L	U>C	AUG	S	His83His
	nsp2	884	O	C>U	ACG	N	Arg27Cys
		913	GR	C>U	CCG	S	Ser36Ser
		1059	O, GH	C>U	ACC	N	Thr85Ile
		1163	GR	A>U	AAU	N	Ile120Phe
		1397	O	G>A	AGU	N	Val198Ile
		1440	L	G>A	GGC	N	Gly212Asp
		1515	V	A>G	CAU	N	His237Arg
		1987	GV	A>G	CAC	S	Ser394Ser
		2416	GH	C>U	ACA	S	Tyr537Tyr
		2480	V	A>G	AAT	N	Ile559Val
		2558	V	C>U	ACC	N	Pro585Ser
	nsp3	2891	L	G>A	GGC	N	Ala58Thr
		3037	O,G,GH,GR,GV	C>U	UCU	S	Phe106Phe
		3177	S	C>U	CCU	N	Pro153Leu
		3267	GR	C>U	ACU	N	Thr183Ile
		4540	S	C>U	ACA	S	Tyr607Tyr
		4543	GH	C>U	CCA	S	Thr608Thr
		5388	GR	C>A	GCU	N	Ala890Glu
		5572	V	G>U	UGU	N	Met951Ile
		5629	GH	G>U	CGU	S	Thr970Thr
		5986	GR	C>U	UCA	S	Phe1089Phe
		6286	GV	C>U	CCU	S	Thr1189Thr
		6310	O	C>A	GCA	N	Ser1197Arg
		6312	O	C>A	ACA	N	Thr1198Lys
		6954	GR	U>C	AUA	N	Ile1412Thr
		7540	GR	U>C	CUA	S	Thr1608Thr
		7926	GV	C>U	GCA	N	Ala1736Val
	nsp4	8782	S	C>U	GCC	S	Ser76Ser
		9223	V	C>U	ACG	S	His223His
		9477	S	U>A	UUU	N	Phe308Tyr
		9526	GH	G>U	UGU	N	Met324Ile
		9745	GV	C>U	ACC	S	Tyr397Tyr
		10319	GH	C>U	GCU	N	Leu89Phe
	nsp5A	10798	G	C>A	ACA	S	Asp248Asp
	nsp6	11083	O,V	G>U	UGU	N	Leu37Phe

		11230	S	G>U	UGC	N	Met86Ile
		11396	GV	C>U	ACU	N	Leu142Phe
		11497	GH	C>U	ACU	S	Tyr175Tyr
		11752	L	C>U	UCC	S	Leu260Leu
	nsp7	11916	GH	C>U	UCA	N	Ser25Leu
ORF1b	nsp12	13730	O	C>U	GCU	N	Ala97Val
		13862	G	C>U	ACA	N	Thr141Ile
		13993	GH	G>U	AGC	N	Ala185Ser
		14408	O,G,GH,GR,GV	C>U	CCU	N	Pro323Leu
		14676	GR	C>U	CCG	S	Pro412Pro
		14805	S,V	C>U	ACU	S	Tyr455Tyr
		15279	GR	C>U	ACC	S	His613His
		15324	G	C>U	ACA	S	Asn628Asn
		15766	GH	G>U	AGU	N	Val776Leu
		16176	GR	U>C	CUU	S	Thr912Thr
	nsp13	16647	GR	G>U	CGC	S	Thr137Thr
		16889	GH	A>G	AAA	N	Lys218Arg
		17019	GH	G>U	AGU	N	Glu261Asp
		17247	V	U>C	GUG	S	Arg337Arg
		17410	L	C>U	ACG	N	Arg392Cys
		17615	GR	A>G	AAG	N	Lys460Arg
		17747	S	C>U	CCU	N	Pro504Leu
		17858	S	A>G	UAU	N	Tyr541Cys
	nps14	18060	S	C>U	UCU	S	Leu7Leu
		18424	GH	A>G	UAA	N	Asp129Asn
		18555	GR	C>U	ACA	S	Asp172Asp
		18736	S	U>C	AUU	N	Phe233Leu
		18877	GH	C>U	UCU	S	Leu280Leu
		19524	O	C>U	UCG	S	Leu495Leu
	nsp15	20268	G	A>G	UAG	S	Leu216Leu
	nsp16	20661	GV	U>C	CUA	S	Ser1Ser
		21255	GV	G>C	CGU	S	Ala199Ala
		21304	GH	C>U	ACG	N	Arg216Cys
S	S	21614	GV	C>U	UCU	N	Leu18Phe
		22227	GV	C>U	GCU	N	Ala222Val
		22346	GV	G>U	UGC	N	Ala262Ser
		22444	GH	C>U	ACC	S	Asp294Asp
		22468	S	G>U	CGU	S	Thr302Thr
		22992	GH,GR	G>A	AGC	N	Ser477Asn
		23063	GR	A>U	TAA	N	Asn501Tyr
		23185	O	C>U	UCA	S	Phe541Phe
		23271	GR	C>A	GCU	N	Ala570Asp
		23401	GR	G>A	AGG	S	Gln613Gln
		23403	O,G,GH,GR,GV	A>G	GAU	N	Asp614Gly
		23604	GR	C>A	CCU	N	Pro681His
		23709	GR	C>U	ACA	N	ThrT716Ile

		23929	O	C>U	ACA	S	Tyr756Tyr
		24034	S	C>U	ACA	S	Asn824Asn
		24334	GV	C>U	GCA	S	Ala924Ala
		24506	GR	U>G	UUC	N	Ser982Ala
		24694	S	A>U	GAA	S	Gly1044Gly
		24914	GR	G>C	AGA	N	Asp1118His
ORF3a	ORF3a	25429	G	G>U	UGU	N	Val13Leu
		25563	O,GH	G>U	AGA	N	Gln57His
		25710	GH	C>U	UCU	S	Leu106Leu
		25907	GH	G>U	GGU	N	Gly172Val
		25979	S	G>U	GGA	N	Gly196Val
		26144	V	G>U	GGU	N	Gly251Val
	M	26729	S	U>C	CUG	S	Ala69Ala
		26735	O,GH	C>U	ACA	S	Tyr71Tyr
		26801	GV	C>G	UCA	S	Leu93Leu
		26876	GH	U>C	UUC	S	Ile118Ile
ORF8	ORF8	27944	GV	C>U	ACC	S	His17His
		27964	GH	C>U	UCA	N	Ser24Leu
		27972	GR	C>U	UCA	Stop	Gln27Stop
		28048	GR	G>U	AGA	N	Arg52Ile
		28077	S	G>C	CGU	N	Val62Leu
		28111	GR	A>G	UAC	N	Tyr73Cys
		28144	S	U>C	UUA	N	Leu84Ser
		28167	S	G>A	GGA	N	Glu92Lys
N	N	28280-28282	GR	GAU>CUA		N,N,N	Asp3Leu
		28311	S,O	C>U	CCC	N	Pro13Leu
		28472	GH	C>U	CCC	N	Pro67Ser
		28657	S	C>U	ACG	S	Asp128Asp
		28688	O	U>C	CUU	S	Leu139Leu
		28836	G	C>U	UCA	N	Ser188Leu
		28854	G,GH	C>U	UCA	N	Ser194Leu
		28863	S	C>U	UCA	N	Ser197Leu
		28869	GH	C>U	CCA	N	Pro199Leu
		28878	S	G>A	AGU	N	Ser202Asn
		28881-28882	O,GR	AGG>AAA		N,S	Arg203Lys
		28883	O,GR	G>C	GGG	N	Gly204Arg
		28932	GV	C>U	GCU	N	Ala220Val
		28975	GH	G>C	UGU	N	Met234Ile
		28977	GR	C>U	UCU	N	Ser235Phe
		29366	GV	C>U	ACC	N	Pro365Ser
		29386	GV	C>U	ACA	S	Asp371Asp
		29399	GH	G>A	GGC	N	Ala376Thr
ORF10		29645	GV	G>U	AGU	Stop	Val30Stop
3'UTR		29868	GR	G>A	UGA		

	29870	GR		C>A	ACA

Boxed - mutation hot spot
nsp - non structural protein
* S - synonymous, N - nonsynonymous substitutions
