# Supplementary material for: Mutational Asymmetries in the SARS-CoV-2 Genome May Lead to Increased Hydrophobicity of Virus Proteins
Source: Genes (Basel). 2021 May 27;12(6):826. doi: 10.3390/genes12060826 (PMC8227412; doi:10.3390/genes12060826)
Supplement: Supplementary file 1 [file genes-12-00826-s001.zip › Table S3.pdf]

Table S3. Mutation spectra determined in different SARS-CoV-2 lineages

clade	relative abundance of substitution type [%]											
	A>C	A>G	A>U	C>A	C>G	C>U	G>A	G>C	G>U	U>A	U>C	U>G	sum
S	0.0	4.3	4.3	0.0	0.0	43.5	8.7	4.3	13.0	8.7	13.0	0.0	100.0
L	0.0	0.0	0.0	0.0	0.0	40.0	40.0	0.0	0.0	0.0	20.0	0.0	100.0
V	0.0	22.2	0.0	0.0	0.0	33.3	0.0	0.0	33.3	0.0	11.1	0.0	100.0
O	0.0	4.8	0.0	9.5	0.0	52.4	14.3	4.8	9.5	0.0	4.8	0.0	100.0
G	0.0	18.2	0.0	9.1	0.0	63.6	0.0	0.0	9.1	0.0	0.0	0.0	100.0
GH	0.0	9.4	0.0	0.0	0.0	56.3	6.3	3.1	21.9	0.0	3.1	0.0	100.0
GR	0.0	6.1	9.1	12.1	0.0	27.3	15.2	9.1	6.1	3.0	9.1	3.0	100.0
GV	0.0	8.3	0.0	0.0	4.2	62.5	0.0	4.2	12.5	0.0	8.3	0.0	100.0
