# Supplementary material for: Mutational Asymmetries in the SARS-CoV-2 Genome May Lead to Increased Hydrophobicity of Virus Proteins
Source: Genes (Basel). 2021 May 27;12(6):826. doi: 10.3390/genes12060826 (PMC8227412; doi:10.3390/genes12060826)
Supplement: Supplementary file 1 [file genes-12-00826-s001.zip › Table S4.pdf]

Table S4. Frequency of different substitutions types in the SARS-CoV-2 lineages and among the virus variants identified in a single individual

substitution	A>C [%]		A>G [%]		A>U [%]		C>A [%]		C>G [%]		C>U [%]		G>A [%]		G>C [%]		G>U [%]		U>A [%]		U>C [%]		U>G [%]		total [%]	
	GL	Pa	GL	Pa	GL	Pa	GL	Pa	GL	Pa	GL	Pa	GL	Pa	GL	Pa	GL	Pa	GL	Pa	GL	Pa	GL	Pa	GL	Pa
nonsynonymous	0.0	0.0	5.9	7.0	2.2	3.0	3.7	2.0	0.0	0.0	20.7	19.0	5.9	14.0	3.7	4.0	10.4	13.0	2.2	2.0	2.2	5.0	0.7	2.0	57.8	71.0
synonymous	0.0	0.0	1.5	0.0	0.7	0.0	0.7	1.0	0.7	0.0	22.2	14.0	1.5	0.0	0.7	1.0	2.2	2.0	0.0	0.0	6.7	6.0	0.0	1.0	37.0	25.0
UTR	0.0	0.0	0.0	0.0	0.0	0.0	0.7	0.0	0.0	0.0	1.5	0.0	0.7	0.0	0.0	1.0	0.7	1.0	0.0	0.0	0.0	0.0	0.0	0.0	3.7	2.0
stop	0.0	0.0	0.0	0.0	0.0	0.0	0.0	0.0	0.0	0.0	0.7	1.0	0.0	0.0	0.0	0.0	0.7	1.0	0.0	0.0	0.0	0.0	0.0	0.0	1.5	2.0
total	0.0	0.0	7.4	7.0	3.0	3.0	5.2	3.0	0.7	0.0	45.2	34.0	8.1	14.0	4.4	6.0	14.1	17.0	2.2	2.0	8.9	11.0	0.7	3.0	100.0	100.0

GL - Global pattern; Pa - virus variants identified in the course of medical treatment of a single individual
