# Supplementary material for: Mutational Asymmetries in the SARS-CoV-2 Genome May Lead to Increased Hydrophobicity of Virus Proteins
Source: Genes (Basel). 2021 May 27;12(6):826. doi: 10.3390/genes12060826 (PMC8227412; doi:10.3390/genes12060826)
Supplement: Supplementary file 1 [file genes-12-00826-s001.zip › Table S5.pdf]

Table S5. Statistical comparisons of SNV in early and late clades of SARS-CoV-2 genomes

mutation	1% treshold early clades		late clades		5% treshold early clades		late clades	
	no	%	no	%	no	%	no	%
A>C	0	0.0	0	0.0	0	0	0	0
A>G	4	5.9	10	8.1	2	10	2	4
A>U	1	1.5	4	3.3	0	0	3	6
C>A	2	2.9	3	2.4	1	5	3	6
C>G	1	1.5	1	0.8	0	0	1	2
C>U	31	45.6	60	48.8	11	55	21	42
G>A	9	13.2	6	4.9	1	5	3	6
G>C	2	2.9	5	4.1	0	0	4	8
G>U	9	13.2	19	15.4	2	10	4	8
U>A	2	2.9	1	0.8	0	0	1	2
U>C	6	8.8	8	6.5	2	10	3	6
U>G	0	0.0	1	0.8	0	0	1	2
indels	1	1.5	5	4.1	1	5	4	8
sum	68	100	123	100	20	100	50	100
Rp	0.9724				0.9630			
Rs	0.8584				0.5896			
P2	0.0002				0.0339			
comment	strong positive correlation				moderate positive correlation			
