# Supplementary material for: Mutational Asymmetries in the SARS-CoV-2 Genome May Lead to Increased Hydrophobicity of Virus Proteins
Source: Genes (Basel). 2021 May 27;12(6):826. doi: 10.3390/genes12060826 (PMC8227412; doi:10.3390/genes12060826)
Supplement: Supplementary file 1 [file genes-12-00826-s001.zip › Table S6.pdf]

Table S6. Amino acids hydrophobicity changes induced by nonsynonymous nucleotide substitutions along the virus genome

Region	Coordinate	Clade(s)	Mutation	amino acids change	character[(1)]	Hydrophobicity changes Method A[(2)] Reference	Allele	Shift	Method B[(2)] Reference	Allele	Shift
ORF1a	490	S	U>A	Asp75Glu	P>P	-3.5	-3.5	0	-123	-106	17
	884	O	C>U	Arg27Cys	P>N	-4.5	2.5	7	-130	78	208
	1059	O, GH	C>U	Thr85Ile	P>N	-0.7	4.5	5.2	-9	172	181
	1163	GR	A>U	Ile120Phe	N>N	4.5	2.8	-1.7	172	179	7
	1397	O	G>A	Val198Ile	N>N	4.2	4.5	0.3	123	172	49
	1440	L	G>A	Gly212Asp	N>P	-0.4	-3.5	-3.1	0	-123	-123
	1515	V	A>G	His237Arg	P>P	-3.2	-4.5	-1.3	-34	-130	-96
	2480	V	A>G	Ile559Val	N>N	4.5	4.2	-0.3	172	123	-49
	2558	V	C>U	Pro585Ser	P>P	-1.6	-0.8	0.8	47	-57	-104
	2891	L	G>A	Ala58Thr	N>P	1.8	-0.7	-2.5	46	-9	-55
	3177	S	C>U	Pro153Leu	P>N	-1.6	3.8	5.4	47	165	118
	3267	GR	C>U	Thr183Ile	P>N	-0.7	4.5	5.2	-9	172	181
	5388	GR	C>A	Ala890Glu	N>P	1.8	-3.5	-5.3	46	-106	-152
	5572	V	G>U	Met951Ile	N>N	1.9	4.5	2.6	119	172	53
	6310	O	C>A	Ser1197Arg	P>P	-0.8	-4.5	-3.7	-57	-130	-73
	6312	O	C>A	Thr1198Lys	P>P	-0.7	-3.9	-3.2	-9	-100	-91
	6954	GR	U>C	Ile1412Thr	N>P	4.5	-0.7	-5.2	172	-9	-181
	7926	GV	C>U	Ala1736Val	N>N	1.8	4.2	2.4	46	123	77
	9477	S	U>A	Phe308Tyr	N>P	2.8	-1.3	-4.1	179	64	-115
	9526	GH	G>U	Met324Ile	N>N	1.9	4.5	2.6	119	172	53
	10319	GH	C>U	Leu89Phe	N>N	3.8	2.8	-1	165	179	14
	11083	O,V	G>U	Leu37Phe	N>N	3.8	2.8	-1	165	179	14
	11230	S	G>U	Met86Ile	N>N	1.9	4.5	2.6	119	172	53
	11396	GV	C>U	Leu142Phe	N>N	3.8	2.8	-1	165	179	14
	11916	GH	C>U	Ser25Leu	P>N	-0.8	3.8	4.6	-57	165	222
ORF1b	13730	O	C>U	Ala97Val	N>N	1.8	4.2	2.4	46	123	77
	13862	G	C>U	Thr141Ile	P>N	-0.7	4.5	5.2	-9	172	181
	13993	GH	G>U	Ala185Ser	N>P	1.8	-0.8	-2.6	46	-57	-103
	14408	O,G,GH,GR,GV	C>U	Pro323Leu	P>N	-1.6	3.8	5.4	47	165	118

	15766	GH	G>U	Val776Leu	N>N	4.2	3.8	-0.4	123	165	42
	16889	GH	A>G	Lys218Arg	P>P	-3.9	-4.5	-0.6	-100	-130	-30
	17019	GH	G>U	Glu261Asp	P>P	-3.5	-3.5	0	-106	-123	-17
	17410	L	C>U	Arg392Cys	P>N	-4.5	2.5	7	-130	78	208
	17615	GR	A>G	Lys460Arg	P>P	-3.9	-4.5	-0.6	-100	-130	-30
	17747	S	C>U	Pro504Leu	P>N	-1.6	3.8	5.4	47	165	118
	17858	S	A>G	Tyr541Cys	P>N	-1.3	2.5	3.8	64	78	14
	18424	GH	A>G	Asp129Asn	P>P	-3.5	-3.5	0	-123	-110	13
	18736	S	U>C	Phe233Leu	N>N	2.8	3.8	1	179	165	-14
	21304	GH	C>U	Arg216Cys	P>N	-4.5	2.5	7	-130	78	208
S	21614	GV	C>U	Leu18Phe	N>N	3.8	2.8	-1	165	179	14
	22227	GV	C>U	Ala222Val	N>N	1.8	4.2	2.4	46	123	77
	22346	GV	G>U	Ala262Ser	N>P	1.8	-0.8	-2.6	46	-57	-103
	22992	GH,GR	G>A	Ser477Asn	P>P	-0.8	-3.5	-2.7	-57	-110	-53
	23063	GR	A>U	Asn501Tyr	P>P	-3.5	-1.3	2.2	-110	64	174
	23271	GR	C>A	Ala570Asp	N>P	1.8	-3.5	-5.3	46	-123	-169
	23403	O,G,GH,GR,GV	A>G	Asp614Gly	P>N	-3.5	-0.4	3.1	-123	0	123
	23604	GR	C>A	Pro681His	P>P	-1.6	-3.2	-1.6	47	-34	-81
	23709	GR	C>U	ThrT716Ile	P>N	-0.7	4.5	5.2	-9	172	181
	24506	GR	U>G	Ser982Ala	P>N	-0.8	1.8	2.6	-57	46	103
	24914	GR	G>C	Asp1118His	P>P	-3.5	-3.2	0.3	-123	-34	89
ORF3a	25429	G	G>U	Val13Leu	N>N	4.2	3.8	-0.4	123	165	42
	25563	O,GH	G>U	Gln57His	P>P	-3.5	-3.2	0.3	-81	-34	47
	25907	GH	G>U	Gly172Val	N>N	-0.4	4.2	4.6	0	123	123
	25979	S	G>U	Gly196Val	N>N	-0.4	4.2	4.6	0	123	123
	26144	V	G>U	Gly251Val	N>N	-0.4	4.2	4.6	0	123	123
ORF8	27964	GH	C>U	Ser24Leu	P>N	-0.8	3.8	4.6	-57	165	222
	28048	GR	G>U	Arg52Ile	P>N	-4.5	4.5	9	-130	172	302
	28077	S	G>C	Val62Leu	N>N	4.2	3.8	-0.4	123	165	42
	28111	GR	A>G	Tyr73Cys	P>N	-1.3	2.5	3.8	64	78	14
	28144	S	U>C	Leu84Ser	N>P	3.8	-0.8	-4.6	165	-57	-222
	28167	S	G>A	Glu92Lys	P>P	-3.5	-3.9	-0.4	-106	-100	6
N	28280-28282	GR	GAU>CUA	Asp3Leu	P>P	-3.5	3.8	7.3	-123	165	288
	28311	S,O	C>U	Pro13Leu	P>N	-1.6	3.8	5.4	47	165	118

28472	GH	C>U	Pro67Ser	P>P	-1.6	-0.8	0.8	47	-57	-104
28836	G	C>U	Ser188Leu	P>N	-0.8	3.8	4.6	-57	165	222
28854	G,GH	C>U	Ser194Leu	P>N	-0.8	3.8	4.6	-57	165	222
28863	S	C>U	Ser197Leu	P>N	-0.8	3.8	4.6	-57	165	222
28869	GH	C>U	Pro199Leu	P>N	-1.6	3.8	5.4	47	165	118
28878	S	G>A	Ser202Asn	P>P	-0.8	-3.5	-2.7	-57	-110	-53
28881-28882	O,GR	AGG>AAA	Arg203Lys	P>P	-4.5	-3.9	0.6	-130	-100	30
28883	O,GR	G>C	Gly204Arg	N>P	-0.4	-4.5	-4.1	0	-130	-130
28932	GV	C>U	Ala220Val	N>N	1.8	4.2	2.4	46	123	77
28975	GH	G>C	Met234Ile	N>N	1.9	4.5	2.6	119	172	53
28977	GR	C>U	Ser235Phe	P>N	-0.8	2.8	3.6	-57	179	236
29366	GV	C>U	Pro365Ser	P>P	-1.6	-0.8	0.8	47	-57	-104
29399	GH	G>A	Ala376Thr	N>P	1.8	-0.7	-2.5	46	-9	-55

(1)Polar (P), nonpolar (NP), no change [0]

(2) Hydrophobicity scale of Kyte and Doolittle (method A) and Palecz (method B)

Polar nonpolar and reverse [NP], no change [0]
Boxed - mutation hot spot
nsp - non structural protein
