# Supplementary material for: Mutational Asymmetries in the SARS-CoV-2 Genome May Lead to Increased Hydrophobicity of Virus Proteins
Source: Genes (Basel). 2021 May 27;12(6):826. doi: 10.3390/genes12060826 (PMC8227412; doi:10.3390/genes12060826)
Supplement: Supplementary file 1 [file genes-12-00826-s001.zip › Table S7.pdf]

Table S7. Comparisons of amino acid hydrophobicity changes between early and late clades, as well as single individual.

group 1					group 2					grop1/group2 comparative statistics				
name	descriptive statistics				name	descriptive statistics				T-test		**Mann whitney**		
	N	average	SD	median		N	average	SD	median	p2	95% level	p2	Z	95% level
early	34	1.2	3.46	0.7	late	48	1.45	3.45	1.5	0.36	not signif.	0.88	-0.16	not signif.
early	34	1.2	3.46	0.7	single ind.	70	0.61	3.1	0.3	0.10	not signif.	0.44	0.77	not signif.
late	48	1.45	3.45	1.5	single ind.	70	0.61	3.1	0.3	0.09	not signif.	0.21	1.25	not signif.

Early and late groups are defined in Table 1
