# Supplementary material for: Mutational Asymmetries in the SARS-CoV-2 Genome May Lead to Increased Hydrophobicity of Virus Proteins
Source: Genes (Basel). 2021 May 27;12(6):826. doi: 10.3390/genes12060826 (PMC8227412; doi:10.3390/genes12060826)
Supplement: Supplementary file 1 [file genes-12-00826-s001.zip › Table S8.pdf]

Table S8. Frequency of nucleotides in motifs flanking the C>U substitutions

	5' position			3' position	
**Base**	**Count [C]**	**Count after normalisation**	**Percentage**	**Count [C]**	**Count after normalisation**
A	25	20.90	31.7	22	18.39
C	10	13.59	20.6	19	25.82
G	7	8.93	13.5	9	11.48
U	29	22.59	34.2	21	16.36
**Sum**	**71**	**66.00**	**100.0**	**71**	**72.04**

Data sets to Table S8

**Triplet**	**Count**	**Frequency**
ACA	9	0.126760563
ACC	7	0.098591549
ACG	5	0.070422535
ACU	4	0.056338028
CCA	2	0.028169014
CCC	2	0.028169014
CCG	2	0.028169014
CCU	4	0.056338028
GCA	2	0.028169014
GCC	1	0.014084507
GCG	0	0
GCU	4	0.056338028
UCA	9	0.126760563
UCC	9	0.126760563
UCG	2	0.028169014
UCU	9	0.126760563
**Sum**	**71**	

Nucleotide content (reference genome)

	Frequency [f(Ni)]
Adenine (A)	0.299
Cytosine (C)	0.184
Guanine (G)	0.196
uracile (U)	0.321
Sum	1

Formula used for normalisation of nucleotide counts:

Count=Ci/(f(Ni)*4)

where: Ci - observed count if i-nucleotide

f(Ni) - frequency of i-nucleotide in the genome
