# Supplementary material for: Mutational Asymmetries in the SARS-CoV-2 Genome May Lead to Increased Hydrophobicity of Virus Proteins
Source: Genes (Basel). 2021 May 27;12(6):826. doi: 10.3390/genes12060826 (PMC8227412; doi:10.3390/genes12060826)
Supplement: Supplementary file 1 [file genes-12-00826-s001.zip › Table S9.pdf]

Table S9. Nucleotide composition in consensus sequences from different clades

character	reference	clade							
	(MN908947)	L	O	S	V	G	GV	GH	GR
counts of A	8876	8876	8876	8876	8876	8875	8875	8875	8877
frequency of A	0.29869	0.29869	0.29869	0.29869	0.29869	0.29866	0.29866	0.29866	0.29873
counts of C	5461	5461	5461	5461	5460	5458	5455	5457	5459
frequency of C	0.18377	0.18377	0.18377	0.18377	0.18374	0.18367	0.18357	0.18364	0.18371
counts of G	5826	5826	5825	5826	5824	5827	5825	5826	5824
frequency of G	0.19606	0.19606	0.19602	0.19606	0.19599	0.19609	0.19602	0.19606	0.19599
counts of U	9553	9553	9554	9553	9556	9556	9561	9558	9556
frequency of U	0.32148	0.32148	0.32151	0.32148	0.32158	0.32158	0.32175	0.32164	0.32158
counts of C+G	11287	11287	11286	11287	11284	11285	11280	11283	11283
frequency of C+G	0.37983	0.37983	0.37980	0.37983	0.37973	0.37976	0.37959	0.37969	0.37969
counts A + U	18429	18429	18430	18429	18432	18431	18436	18433	18433
frequency of A + U	0.62017	0.62017	0.62020	0.62017	0.62027	0.62024	0.62041	0.62031	0.62031
length [bp]	29716	29716	29716	29716	29716	29716	29716	29716	29716
